# Carbon nanodots interference with lactate dehydrogenase assay in human monocyte THP-1 cells

**DOI:** 10.1186/2193-1801-3-615

**Published:** 2014-10-18

**Authors:** Petra C Wright, Hu Qin, Martin MF Choi, Norman HL Chiu, Zhenquan Jia

**Affiliations:** Department of Chemistry and Biochemistry, University of North Carolina at Greensboro, Greensboro, NC 27412 USA; Department of Chemistry, Hong Kong Baptist University, Kowloon Tong, Hong Kong, SAR China; Department of Nanoscience, Joint School of Nanoscience and Nanoengineering, Greensboro, NC 27401 USA; Department of Biology, University of North Carolina at Greensboro, Greensboro, NC 27412 USA

**Keywords:** Carbon nanodots, Interference, Lactate dehydrogenase assay, Trypan blue staining, Cytotoxicity

## Abstract

**Background:**

Carbon nanodots (CD), a new class of carbon nanomaterials with sizes below 10 nm, have recently attracted wide attention due to their superiority in water solubility, chemical inertness, and resistance to photobleaching. As a result, CD has found important and wide applications in energy, catalysis, biological labeling, bioimaging and drug delivery. On the other hand, due to the lack of available toxicity data, there is a growing concern regarding the potential risks of CD. Hence, accurate assessment of the cytotoxicity of CD has become more important than ever before. The lactate dehydrogenase (LDH) assay is widely used to detect cytotoxicity of various nanoparticles including CD. Many recent studies used LDH assay to study the CD toxicity in various cells. However, these studies failed to further examine whether the CD were interfering with the LDH assay which would alter their findings.

**Findings:**

This study investigated the possible interference of carbon nanodots on the LDH assay in human monocyte THP-1 cells. Monocytes are known to be involved in inflammable vascular diseases, and have been suggested to be the targets for CD exposure. In this study, the cytotoxicity of CD in concentrations ranging from 0.075 to 0.60 mg/mL, was determined by using the LDH assay. To validate the results of LDH assay, the cell counting method with trypan blue staining was used. With 24 hours incubation time, the cell viability of THP-1 was significantly decreased according to the trypan blue staining method. Whereas, in the LDH assay, the CD was found to interfere in a dose-dependent manner with the NADH absorbance measurements at 340 nm.

**Conclusions:**

This study represents the first report on the negative interference of CD on LDH assay, and caution should be observed when evaluating the cytotoxicity of CD.

## Introduction

Carbon nanomaterial production has increased globally in the past few years and risk assessment for carbon nanomaterials is not completely established (Tsuji et al. 2006). Their physical and chemical properties allow them to be great candidates for imaging, photocatalysis, cancer cell inhibition, and disease diagnosis (Hsu et al. 2013; Juzenas et al. 2013; Tang et al. 2013; Zhang et al. 2013). Carbon nanodots (CD), a new class of carbon nanomaterials with sizes below 10 nm, have recently attracted wide attention due to their superiority in water solubility, chemical inertness, and resistance to photobleaching (Baker and Baker 2010; Hsu and Chang 2012; Shi et al. 2011; Zhou et al. 2012). As a result, CD has found important and wide applications in energy, catalysis, biological labeling, bioimaging and drug delivery (Baker and Baker 2010; Hsu and Chang 2012; Shi et al. 2011; Zhou et al. 2012). Although CD are considered to be biocompatible, there have been cases where CD is identified as toxic agent for various biological tissues (Hsu et al. 2013; Zhang et al. 2013). Therefore, it is important that the risk assessment is thoroughly examined before administering them for medical use. The correlation between exposure to nanoparticles and vascular diseases is of particular concern (Mossman et al. 2007; Beck and Offenbacher 2001). Monocytes are known to play crucial role in the development of inflammable vascular diseases and have been suggested to be significant targets for nanoparticle exposure (Prach et al. 2013).

Although there are different types of assays that can be applied to evaluate the cytotoxicity of nanomaterials, lactate dehydrogenase (LDH) assay has been considered to be the gold standard for measuring cytotoxicity (Kroll et al. 2012). The LDH enzyme is shared amongst all cell types and is released upon damage to the cell membrane. The LDH assay has been used to access the properties of CD, as a way to support CD induces apoptosis instead of necrosis (Hsu et al. 2013). Hsu et al., measured the integrity of the cell membranes of three different cancer cell lines MCF-7, MDA-MB-231, and HeLa by using the LDH assay. They found that after 24 h incubation with CD there wasn’t any increase in the release of LDH in any of the selected cell lines (Hsu et al. 2013). However, they did not further examine whether the CD were interfering with the LDH assay which would alter their findings. This study focuses on the investigation of possible interference of carbon nanodots on the LDH assay in human monocyte THP-1 cells.

## Methods

### Synthesis and characterization of carbon nanodots

Carbon nanodots were synthesized as reported recently by us (Hu et al. 2013). Briefly, 0.50 g citric acid (CA) was dissolved in 5.0 mL distilled deionized (DDI) water followed by mixing with various amounts of 1,2-ethylenediamine (EDA, 0.00–0.47 g) under vigorous stirring in glass vials. The solution was then heated in a domestic microwave oven (800 W) for 4.0 min and the glass vial was cooled down to room temperature. The reddish brown and foamy solid was then dissolved in DDI water and dialyzed through a dialysis membrane with MWCO of 500–1000 Da (Spectrum Laboratories, Rancho Dominguez, CA, USA) and a clear and reddish brown aqueous solution was lyophilized to obtain *ca.* 0.050 g dry C-dots product. Various optical determinations including UV–vis absorption, photoluminescence spectroscopy, IR spectroscopy and mass spectrum were employed to characterize the as-synthesized C-dots as described by us recently (Hu et al. 2013).

### Cell culture and treatment

The human monocyte THP-1 cells (ATCC, Manassas, VA) were maintained in RPMI-1640 medium supplemented with fetal bovine serum (10%), penicillin-streptomycin (1%) antibiotic in 5% CO_2_ at 37°C. All these chemicals and media were purchased from Sigma-Aldrich (St Louis, MO). Prior to confluence, the cells were collected and centrifuged for 10 min, 4°C, 1000 g. The supernatant was decanted and the cell pellet was re-suspended in Dulbecco’s Modified Eagle’s medium (DMEM) The cells were then seeded in a 24-well plate at a density of 4.0 × 10^5^ cells per mL. CD (0.60 mg) were mixed with DMEM (1 mL), an aliquot (400 uL) of this mixture was added to each well, and was incubated for 24 h.

### LDH assay

The cytotoxic effects of CD were first measured by quantitating the release of lactate dehydrogenase (LDH) from the THP-1 cells. Following incubation, an aliquot (200 uL per well) of treated cells were centrifuged for 5 min, 4°C, 13,000 × g. The untreated cells (200 uL per well) were sonicated then centrifuged. The supernatants were collected for LDH measurements. Reagents for LDH assay were 60 uL per well of 0.8 mg/mL pyruvate, 60 uL per well of 3 mg/mL NADH. A total assay volume of 600 uL was made up with 1X phosphate buffered saline (PBS, pH 7.4). NADH was added last, and the cuvette was immediately placed in the spectrophotometer. In the spectrophotometer, the oxidation of NADH was monitored at 340 nm for over 5 min.

### Trypan blue viability assay

The cell number and cell viability were determined by using the cell counting method. Cells (100 uL per well) were mixed with trypan blue dye (100 uL) and 20 uL of this cell-dye mixture was loaded onto a hemacytometer. Each mixture was counted four times by using all four grids on the hemacytometer. When digital EVOS microscope was used, an aliquot of 10 uL of the cell-dye mixture was added on a microscope slide.

### Measurement of CD absorbance with a spectrophotometer

The CD (0.15 mg) was dissolved in deionized water (0.25 mL) for absorbance measurements. Serial dilutions were performed for concentrations of 0.45, 0.30, 0.15, 0.075 mg/mL. Each CD dilution (10 uL) was added to the LDH reagents (60 uL NADH, 60 uL pyruvate, and 490 uL 1X PBS), and absorbance was read at 340 nm.

### Statistical analyses

Data were analyzed with one-way ANOVA and are expressed as mean ± SEM based on quadruple and triplicate observations, respectively. After ANOVA, statistical significance between the treatment and control groups was determined by Student t-test and defined at p < 0.05 level.

## Results and discussion

**Figure 1:**
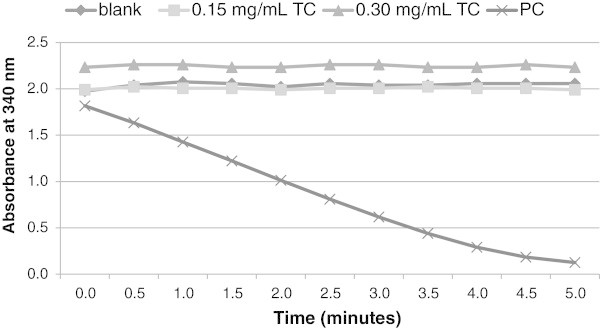
**Results of LDH assays of THP-1 cells with or without CD.** Cells were treated without or with 0.15 mg/mL and 0.30 mg/mL CD for 24 h. Blank contained PBS buffer only. Supernatant from treated cells (TC) was measured by mixing 5 or 10 uL of supernatant with 595 or 590 uL of LDH reagents, respectively. Positive control (PC) consisted of lysate from control cells in the absence of CD.

Recent studies have demonstrated that exposure to nanoparticles could elevate the risk of vascular diseases. Monocytes are known to be involved in inflammable vascular diseases, and have been suggested to be the targets for nanoparticle exposure. In this study, the cytotoxicity of monocytes, after exposure to CD in concentrations ranging from 0.075 to 0.60 mg/mL, was determined by using the LDH assay. Figure 1 displays the LDH absorbance in the presence and absence of CD. There was a pronounced effect of LDH release in the cell lysate (Figure 1, positive control) indicating LDH was utilizing NADH in its conversion to pyruvate. The decrease in NADH absorbance over time confirmed that the reagents and instrument were operating properly. However, after 24 h incubation with 0.15 mg/mL and 0.30 mg/mL of CD, the CD did not cause any increase in the release of LDH from the THP-1 cells (Figure 1).

**Figure 2:**
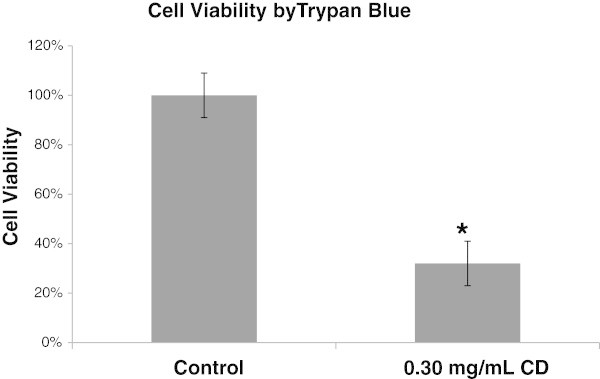
**Cell viability of THP-1 cells determined by cell counting with Trypan blue staining method with or without CD.** The percentage of viable cells was normalized to the negative control, which was calculated by multiplying the ratio of (number of living cells in treated sample: number of living cells in negative control) with 100%. Data are expressed as mean ± SEM from four experiments. *, p < 0.05 vs. control.

**Figure 3:**
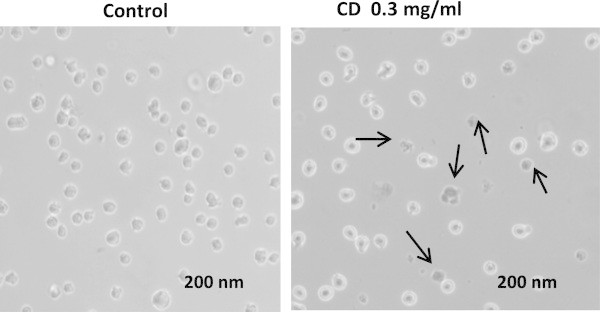
**Morphology change of THP-1 cells in absence and presence of CD.** There are distinct changes between the negative control and the treated cells. The arrows show that the cells have significant changes in cell morphology.

To validate the results of LDH assay, the cell counting method with trypan blue staining was used (Figure 2). Incubation of cells with various concentrations of CD for 24 hours caused a significant decrease in cell viability measured by trypan blue staining (Figure 2). The cell morphology changes by CD were further examined with a digital microscope (Figure 3). In comparison to the control, the cells treated with CD display significant changes in cell morphology including a loss of uniformity and detritus surrounding the cell clusters as indicated by the arrows in Figure 3. The inconsistency results between Figure 1 (LDH assay) and Figures 2 and 3 (trypan blue staining and digital microscope) by CD treatments lead us to suspect CD could interfere with the NADH absorbance measurements in the LDH assay (Figure 1).

**Figure 4:**
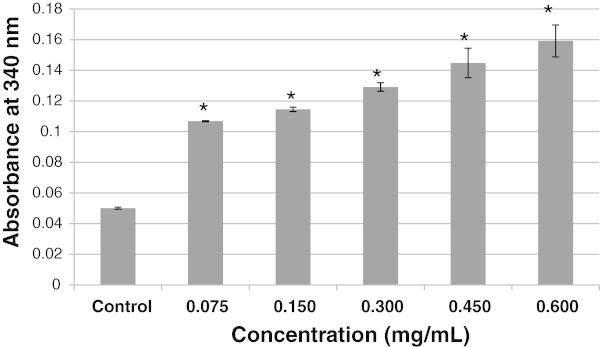
**Absorbance of CD at 340 nm.** The reported values (mean ± SD) were corrected by subtracting the background absorbance from PBS buffer. Data are expressed as mean ± SEM from three experiments. *, p < 0.05 vs. control.

The absorbance of CD at 340 nm was measured to confirm its interference with the LDH assay (Figure 4). The absorbance of CD in the absence of THP-1 cells was measured in concentrations ranging from 0.075 to 0.60 mg/mL. The absorbance of CD at 340 nm increased as the concentrations increased (R^2^ = 0.9883, Figure 4). This trend was observed in the LDH assay, suggesting that there was interference on the NADH absorbance (340 nm) by the CD.

Although the LDH assay is widely used to detect cytotoxicity of various nanoparticles including CD, there is limited information on the possible interference of LDH on carbon nanodots (CD). Recently, Hsu et.al reported that the observed cytotoxicity of carbon nanodots did not increase the release of LDH following 24 h incubation of MCF-7, MDA-MD-231 and HeLa cells with CD concentrations ranging from 0.075 to 0.60 mg/mL (Hsu et al. 2013). However, the authors failed to further examine whether CD was interfering with the LDH assay which might alter their conclusion. In this study, we are the first to report that CD was found to interfere with the NADH absorbance measurements in the LDH assay in a dose-dependent manner. Our results suggest that LDH assay should not be used to evaluate the cytotoxicity of CD that potentially leads to a false conclusion on the cytotoxicity of carbon nanodots as reported by Hsu et.al (Hsu et al. 2013). The traditional trypan blue staining method proved to be a viable approach for evaluating the cytotoxicity of nanomaterials, whose chemical and physical properties may not be known. This method has been used to access the cytotoxicity of multi-walled carbon nanotubes, however it is fairly time consuming. Therefore, an improved method is needed to determine the risk assessment of CD for any future development or application.

## Abbreviations

CA: Citric acid
CD: Carbon nanodots
DMEM: Dulbecco’s Modified Eagle’s medium, EDA, 1,2-ethylenediamine
LDH: The lactate dehydrogenase
PBS: Phosphate buffered saline
PC: Positive control.
